# A Scalable, High-Efficiency, Low-Energy-Spread Laser Wakefield Accelerator Using a Tri-Plateau Plasma Channel

**DOI:** 10.34133/research.0396

**Published:** 2024-08-09

**Authors:** Shuang Liu, Fei Li, Shiyu Zhou, Jianfei Hua, Warren B. Mori, Chan Joshi, Wei Lu

**Affiliations:** ^1^ Department of Engineering Physics, Tsinghua University, Beijing 100084, China.; ^2^ University of California Los Angeles, Los Angeles, CA 90095, USA.; ^3^ Institute of High Energy Physics, Chinese Academy of Sciences, Beijing 100049, China.; ^4^ Beijing Academy of Quantum Information Science, Beijing 100193, China.

## Abstract

The emergence of multi-petawatt laser facilities is expected to push forward the maximum energy gain that can be achieved in a single stage of a laser wakefield acceleration (LWFA) to tens of giga-electron volts, which begs the question—is it likely to impact particle physics by providing a truly compact particle collider? Colliders have very stringent requirements on beam energy, acceleration efficiency, and beam quality. In this article, we propose an LWFA scheme that can for the first time simultaneously achieve hitherto unrealized acceleration efficiency from the laser to the electron beam of >20% and a sub-1% energy spread using a stepwise plasma structure and a nonlinearly chirped laser pulse. Three-dimensional high-fidelity simulations show that the nonlinear chirp can effectively mitigate the laser waveform distortion and lengthen the acceleration distance. This, combined with an interstage rephasing process in the stepwise plasma, can triple the beam energy gain compared to that in a uniform plasma for a fixed laser energy, thereby dramatically increasing the efficiency. A dynamic beam loading effect can almost perfectly cancel the energy chirp that arises during the acceleration, leading to the sub-percent energy spread. This scheme is highly scalable and can be applied to petawatt LWFA scenarios. Scaling laws are obtained, which suggest that electron beams with parameters relevant for a Higgs factory could be reached with the proposed high-efficiency, low-energy-spread scheme.

## Introduction

In the past 4 decades, the field of laser wakefield acceleration (LWFA) has witnessed numerous milestones [1–8]. The extremely high acceleration gradient and microscopic electromagnetic field structure of an LWFA can provide compact and cost-effective accelerators for high-energy physics research, advanced light sources, imaging using electrons and radiation [9,10], and many other research and practical applications. Emerging multi-petawatt laser projects worldwide and the expected increases in repetition rate and thereby average power of these lasers will provide a further impetus for LWFA to achieve its ultimate scientific goal, a next-generation high-energy particle collider. The principal challenges in realizing even a proof-of-concept LWFA-based compact collider are the simultaneous demands for >100-GeV beam energy, tens of percent of energy transfer efficiency from the drive laser pulse to the accelerating beam, and ultra-high beam quality (ultra-low transverse emittance, <1% root mean square energy spread, and nanocoulomb of charge). Despite the fact that impressive progress has been made in LWFA experiments thus far, these advances have invariably focused on maximizing the highest energy (presently at <10 GeV), which is a necessary but not sufficient requirement to build a future collider based on this paradigm changing technology. An accelerator based on such limited energy gain in a single stage necessitates multi-stage operation to reach energies of interest to particle physics and energy recovery of the unspent laser beam. There are of course other challenges such as generating spin-polarized beams and an overall repetition rate needed to achieve the necessary luminosity in the collision (interaction) volume. Furthermore, similar considerations need to be addressed for the positron arm of a future e-e+ collider. While a comprehensive design of an LWFA-based e-e+ collider is beyond the scope of any single article, it is possible to address issues for the electron arm. In this article—using particle-in-cell (PIC) code simulations and scaling laws—we show that it is possible to design a single 100-GeV high-efficiency LWFA stage that can provide collider-like beam quality using petawatt-class lasers that are rapidly coming online around the world. The resulting electron beam would already meet the requirements for the electron arm of a Higgs factory in terms of beam quality, average energy, and charge [11,12].

In the beam-driven plasma-based acceleration concept, often referred to as plasma wakefield acceleration (PWFA), energy transfer efficiencies from the drive beam to the trailing beam of 60% have been demonstrated in simulations [13,14] and high-quality, high-efficiency acceleration observed in experiments [8,15,16]. However, for an LWFA stage, simulations and experiments have not demonstrated an energy transfer efficiency from the laser pulse to the accelerated electron beam beyond a few percent. Such low acceleration efficiencies of LWFAs primarily stem from the laser pulse shape distortion that occurs because of group velocity dispersion that downshifts frequency of the photons (photon deceleration) and dephasing between the accelerating beam and the continuously temporally evolving laser pulse. Specifically, the nonuniform density and relativistic mass of background electrons in a plasma wake make the photon deceleration rate vary along the laser, leading to longitudinal and transverse pulse distortion before substantial pump depletion can occur. Second, the group velocity of the evolving laser and the resulting wake’s phase velocity are lower than the speed of relativistic electron bunches. Therefore, there is significant phase slippage between accelerated particles and the wake well before pump depletion of the driver occurs. Moreover, the accelerated beam often has a large correlated energy spread (energy chirp) due to the nonnegligible bunch length relative to the (millimeter-scale) accelerating field structure. Methods have been proposed to reduce the energy spread [17–22] and mitigate dephasing [23–31] and pulse depletion [32–34], but a comprehensive proposal is still lacking to simultaneously achieve high overall efficiency, low energy spread, and emittance preservation of an LWFA stage.

**Fig. 1.**
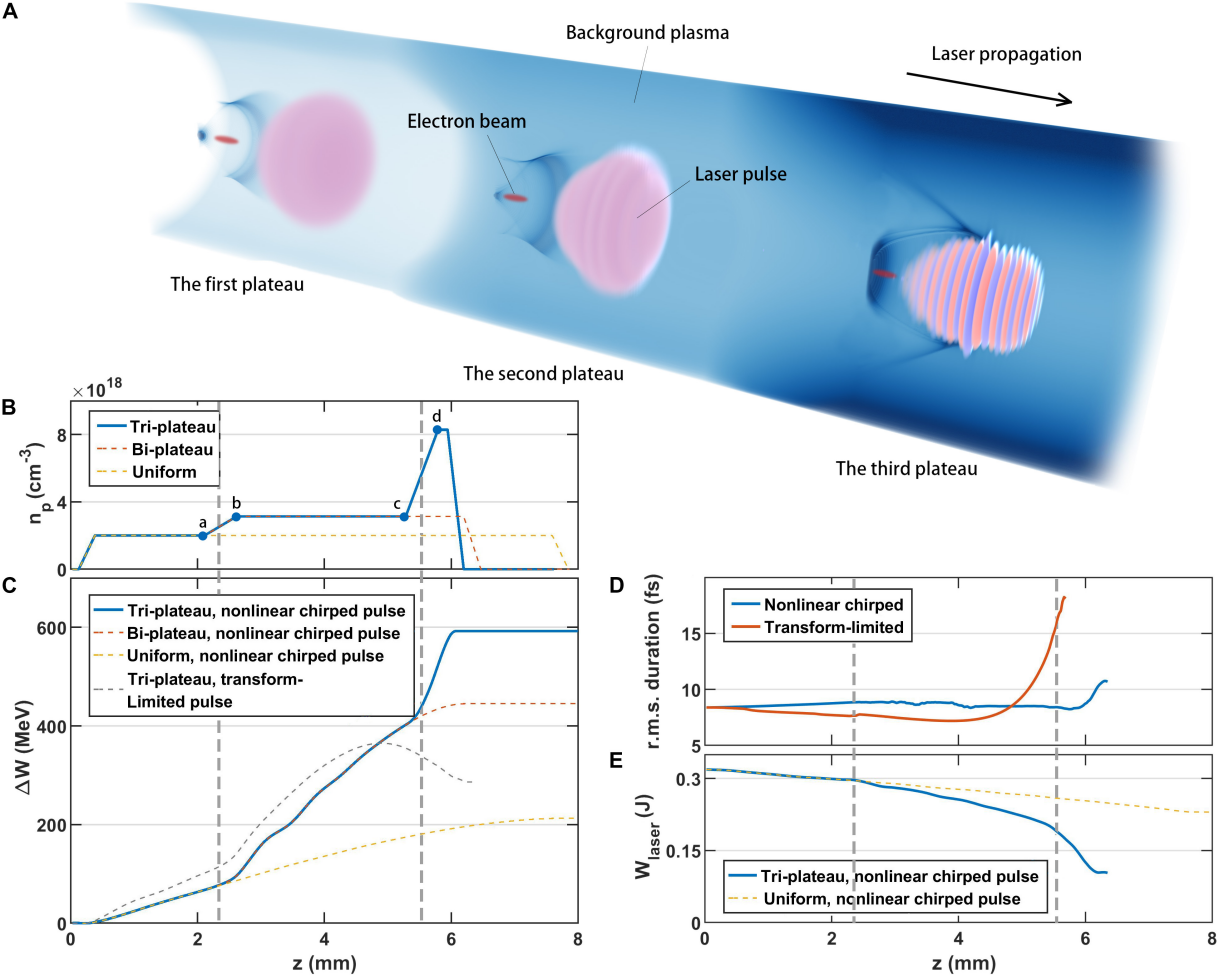
Schematic of the high-efficiency LWFA scheme in the tri-plateau plasma channel. (A) Evolution of laser wakefield in the tri-plateau channel structure (this figure is generated using VisualPIC [56]). (B) On-axis density distribution of the tri-plateau structure. (C) Averaged energy gain in different cases. (D) Pulse length [root mean square (r.m.s.)] evolution of the nonlinear chirped pulse (blue line) and the transform-limited pulse (red line) in the tri-plateau structure. (E) Energy consumption of the nonlinear chirped laser pulse in a uniform plasma channel and the tri-plateau structure.

In this article, we propose a novel scheme that combines a nonlinearly chirped driving laser pulse with a tri-plateau density structure. By using a nonlinearly chirped laser pulse, the axial laser distortion can be significantly mitigated over much longer propagation distance [35,36], leading to a more stable LWFA stage with high laser to wake conversion efficiency. The tri-plateau density structure has 3 axially uniform sections of progressively higher density connected by up-ramps, combining with radially parabolic plasma channels (see Fig. 1). The use of such a properly designed plasma structure can significantly mitigate the dephasing effect and thereby increase the energy gain and overall efficiency. These 2 ideas together lead to energy gains 3 times larger compared with a single uniform density LWFA. At the same time, we discovered that a dynamic beam loading (DBL) effect [37], where the loaded wake initially induces a correlated energy spread that can then be naturally removed as the beam loading effect changes during the wake propagation, leads to accelerated beams with extremely low energy spreads (<1%). The cumulative DBL effect is fully controllable, and the optimal output energy spread can be achieved by tuning the density and length of each density plateau. Furthermore, simulations have confirmed that the proposed scheme is highly scalable. For tens to hundreds of terawatt drive laser scenarios, the scheme can output 0.6- to 10-GeV electron beams with equally high energy efficiency and low energy spread. Scaling the scheme to the petawatt laser-driven regime is expected to result in a remarkable 100-GeV energy gain of a 2-nC electron beam.

## Results and Discussion

### The high-efficiency, low-energy-spread LWFA scheme

We rely on high-fidelity simulations using OSIRIS to illustrate this high-laser-coupling-efficiency, low-energy-spread concept. In Fig. 1A, a 125-pC witness electron beam is accelerated through a tri-plateau density profile to ~600 MeV in a wake driven by a laser pulse with only 340-mJ energy. The resulting energy transfer efficiency from the laser to the electron beam is >21%. High efficiency is achieved for relatively low laser normalized vector potential $a_{0}\equiv \frac{eA_{0}}{m_{e}c^{2}}$ in the blowout regime [38,39]. According to the scaling law [40], the beam energy gain is $E_{b}\propto a_{0}^{5/2}$ and the laser pulse energy is $E_{l}\propto a_{0}^{7/2}$ (assuming a round laser pulse *cτ* ∼ *w*_0_), and the energy transfer efficiency is thus $\Gamma ∼\frac{E_{b}}{E_{l}}∼a_{0}^{-1}$. Here, we chose *a*_0_ = 1.67 to ensure the excitation of plasma wake in the blowout regime and a relatively high energy transfer efficiency. The resulting laser power is not sufficient for self-guiding [41], so a tri-plateau plasma structure with a parabolic transverse density of the form $n_{p}\left(r\right)=n_{p0}\left(1+\frac{\Delta nr^{2}}{w_{0}^{2}}\right)$ is used, where Δ*n* = 0.5 such that the laser with a spot size *w*_0_ is free of diffraction throughout the 6-mm-long plasma. Because the density of each plateau increases in discrete steps (Fig. 1B), the size of the wake cavity shrinks as it transits from one stage to the next stage (Fig. 1A). The on-axis densities, *n*_*p*0_, of the 3 plateaus are 2 × 10^18^ cm^−3^, 3.13 × 10^18^ cm^−3^, and 8.3 × 10^18^ cm^−3^, and the corresponding lengths of plateau are 1.73, 2.67, and 0.15 mm, respectively. Adjacent plateaus are connected by a linear up-ramp of 0.5 mm, and the slopes at both the entrance and the exit for the first and last plateau are 0.25 mm long. Most of the energy gain occurs in the uniform density plateau regions—in this sense, the exact lengths of the density up-ramps between the stages are not critical. The laser pulse has a Gaussian transverse profile, focused to a beam waist *w*_0_ = 10.7 μm at the midpoint of the density up-ramp of the first plateau, and a sin^2^-shaped temporal envelope of the field with a 21.5-fs full width at half maximum (FWHM) pulse length. The laser pulse is nonlinearly chirped to partially compensate the nonlinear dispersion (see Materials and Methods). For ease in interpretation, a bi-flattop electron beam with 3.76-μm beam length is initialized behind the drive laser with an initial energy of 51.1 MeV (*γ* = 100) and 0.1% energy spread, corresponding to the situation of external injection or staging. The resulting beam energy is insensitive to the specific longitudinal profile for a Gaussian-like laser pulse, and >21% energy efficiency with <1% energy spread can be achieved. The initial emittance is 0.1 mm·mrad, and the beam is focused to the midpoint of the first up-ramp with a spot size of 0.5 μm. Compared with the uniform (the yellow line in Fig. 1B) and bi-plateau (the red lines in Fig. 1B) channel structure, the final energy gain of the tri-plateau structure is almost tripled, significantly increasing the acceleration efficiency. The length of the uniform plasma is 8 mm, and the lengths of the 2 plateaus for the bi-plateau are 1.73 and 2.92 mm, respectively. Numerical parameters are given in Materials and Methods.

Because ultra-relativistic electrons essentially move at the speed of light, the accelerated e-beam will gradually outrun the accelerating phase of the plasma wake excited by lasers (dephasing). This causes the acceleration gradient felt by the electron beam to decrease or even become negative as the laser propagates in a uniform plasma. Since the dephasing length is typically much shorter than the pump depletion length, the acceleration gradient will be significantly reduced far before the laser energy is depleted, thus greatly limiting the acceleration efficiency. Due to this dephasing process, the spatial gradient of the energy gain in the laboratory frame gradually reduces and appears parabolic as depicted by the yellow dashed lines in Fig. 1C. On the other hand, during the acceleration process, the laser loses energy in a nearly linear manner as shown by the yellow dashed lines in Fig. 1E, and eventually, only one-third of the initial energy is transferred to the plasma wake. In our tri-plateau scheme, a rephasing process occurs in both the density up-ramp and the higher density plateau that follows it. In the rephasing process, the wavelength of the plasma wake shrinks due to the increase of the plasma density and e-beam finds itself once again close to the maximum acceleration phase, significantly improving the acceleration efficiency. Since the acceleration gradient also increases with approximately square root of the density, the slope of the gain curve (blue line) increases sharply from one plateau to the next. As shown in Fig. 1C, the energy gain of each stage is insensitive to the plasma density, albeit the acceleration distance (being proportional to the dephasing length) becomes shorter as the density increases. Using the scaling laws in the blowout regime [40], for the accelerating field, $E_{z}\propto n_{p}^{1/2}a_{0}^{1/2}$, and the dephasing length, $L_{d}\propto n_{p}^{-1}$, the energy gain scales as $\Delta E\propto n_{p}^{-1/2}a_{0}^{1/2}$. Due to the focusing and steepening of the laser pulse, *a*_0_ increases so that Δ*E* does not change much as *n_p_* increases. As a result, the energy gain is nearly tripled compared with that of the uniform plasma case.

As shown by the blue solid line in Fig. 1C, the energy gain curve now increases almost linearly in each stage, and hence, a high beam loading efficiency is maintained continuously for the 125-pC beam charge. The beam loading efficiency (wake-to-beam efficiency), defined as the ratio of energy extracted by the particles from the wake to the energy cost of the laser, is calculated by *N*〈∆*W*〉/(*W_i_* − *W_o_*) in each plateau, where *N* is the electron number of the beam, 〈∆*W*〉 is the averaged energy gain of the electron beam in this plateau, and *W_i_*/*W_o_* is the laser energy entering/exiting this plateau. In the first 2 plateaus, the wake-to-beam efficiency is 49.6% and then drops to 22.3% in the third plateau. More laser energy is utilized in the tri-plateau plasma; 67.5% of the laser energy is consumed in the proposed scheme, while only 34.0% is consumed in a single uniform plasma channel. The total efficiency is the product of how much of the laser energy is transferred to the wake (roughly the laser energy utilization percentage) times the beam loading efficiency, which is >30% in this case. As a result, a total efficiency of >21% is obtained, which is the highest value achieved to date for a fully self-consistent LWFA concept.

**Fig. 2.**
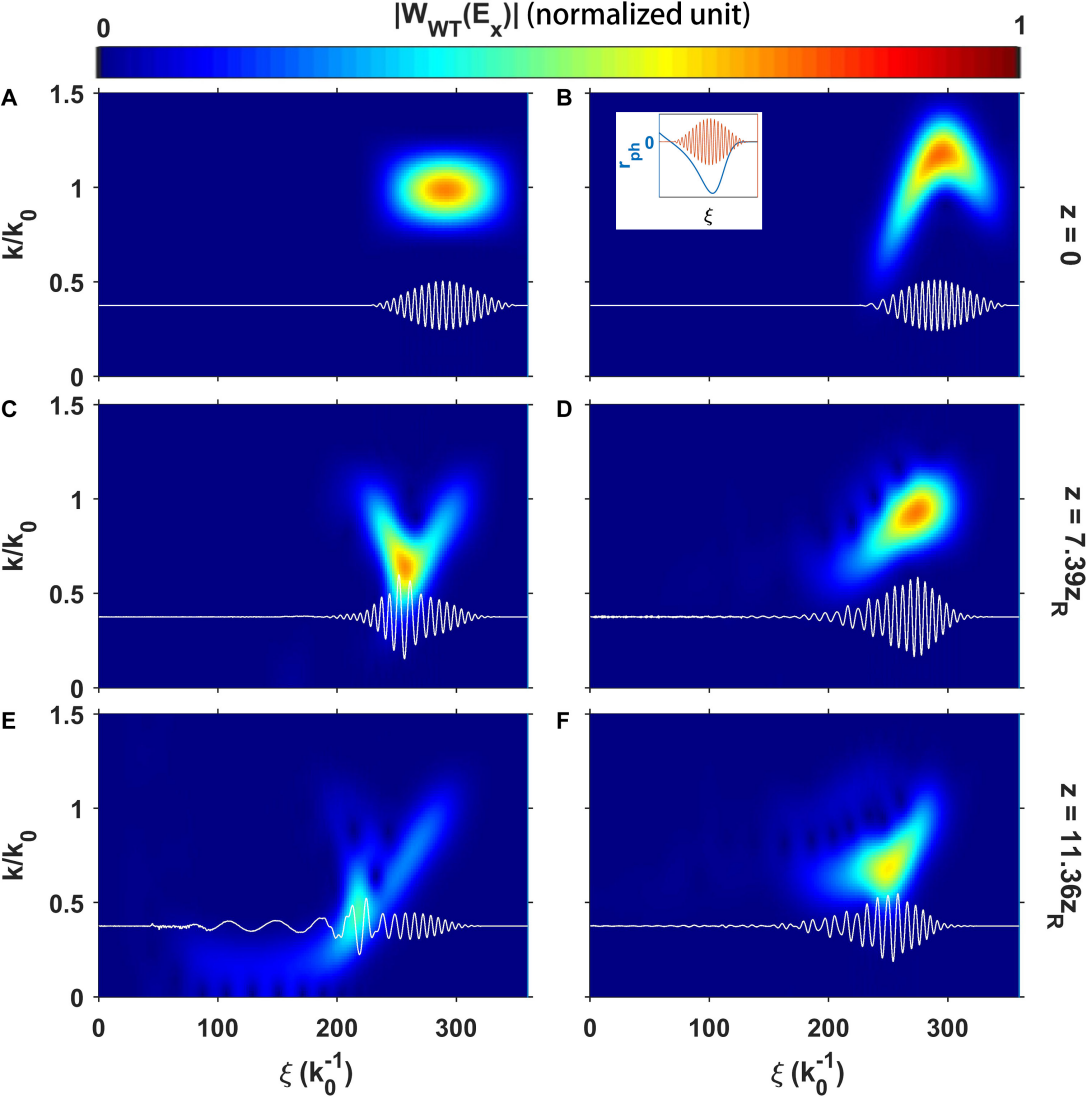
On-axis spectrograms (in color-obtained by Wigner-wavelet transforming the electric field) of the laser pulse at various times for an initially transform-limited laser pulse (A, C, and E) and a chirped laser pulse (B, D, and F) propagating through a single plateau. (A), (C), and (E) show the spectrograms of the original unchirped or transform-limited laser pulse at *z* = 0, *z* = 7.39*z_R_*, and *z* = 11.36*z_R_*, where the Rayleigh length *z_R_* of the original laser pulse is 447 μm. The inset in (B) shows the laser field *E_x_* by the red line and the frequency shift rate by the blue line. As a comparison, (B), (D), and (F) show the spectrograms of the initially chirped laser pulse at the same distances. The spectrograms in all the subplots are obtained by carrying out wavelet transform of *E_x_*, i.e., $\left|W_{\mathrm{WT}}\left(E_{x}\right)\right|=\left|\int_{\xi -\frac{\delta }{2}}^{\xi +\frac{\delta }{2}}E_{x}\left(\xi^{\prime }\right)e^{\mathrm{ik}\xi^{\prime }}{\mathrm{d\xi}}^{\prime }\right|$ with the width of the transform window *δ* = 25.4 μm. The white lines are the on-axis lineouts of *E_x_*. The initially chirped laser pulse is able to maintain an overall similar pulse envelope shape (F) as it has at the beginning (B), whereas the initially unchirped pulse spreads rapidly due to photon deceleration and group velocity dispersion.

We note that, in this proposed scheme, the laser energy is efficiently utilized because the pump laser pulse has a nonlinear chirp with frequency increasing from the front to the middle and then decreasing from the middle to the back, allowing the pump waveform to self-compensate for photon deceleration due to wake formation and maintain its shape. The nonlinear frequency chirp is of the form $\frac{k\left(\xi \right)}{k_{0}}=c_{0}+c_{1}\xi +c_{2}\xi^{2}$, where *k*(*ξ*) and *k*_0_ are the local and central wave number and *ξ* ≡ *ct* − *z*. The chirp coefficients c_0_, c_1_, and c_2_ are 1.2, 1.79 × 10^−3^*k*_0_, and $-\;9.7\times 10^{-5}k_{0}^{2}$, respectively. Such a nonlinear chirp can be realized by either nonlinear cross-phase modulation [42] or nonlinear pulse compression by customized gratings [43,44]. In the blowout regime [38,39], the driving laser pulse with the optimal pulse length resides at the first half of the ion cavity where the photon deceleration rate *r_ph_* < 0 (see Materials and Methods). The occurrence and effects on pulse distortion of photon deceleration/pump depletion are illustrated in Fig. 2. The left column frames are taken from a simulation with a transform-limited pulse, while the right column is for a laser with a nonlinear frequency chirp. For these examples, a laser with identical parameters as above is sent through a single plateau with *n*_*p*0_ = 3 × 10^18^cm^−3^ and Δ*n* = 0.5. For each case, the axial lineout and spectrogram of the laser are shown at propagation distances, 0, 3.81, and 5.08 mm, respectively. Initially, the middle of the pulse experiences a more intense photon deceleration than the head and tail of the pulse (see the inset in Fig. 2A). This can be seen from Fig. 2A, C, and E, where the on-axis spectrogram for the transform-limited laser evolves into a V shape, indicating as expected that the middle part suffers from a faster photon deceleration. The pulse is significantly lengthened as a result of the resulting envelope distortion (Fig. 2E). On the other hand, as shown in Fig 2B, D, and F, the initial nonlinear chirp nearly perfectly balances the redshifting, and the waveform is well preserved throughout the simulation. Off-axis evolution of the spectrogram has a similar pattern shown by Fig. 2, which is supplied in Materials and Methods.

Such a pulse can propagate much further than the pump depletion limit of a transform-limited pulse of similar pulse width and peak intensity. The importance of this chirp is seen in the gray line of Fig 1C, where it can be seen that no energy gain arises in the third stage without the chirp (transform-limited pulse). The reason for saturation of the energy gain can be seen in Fig. 1D to be that the pulse length increases rapidly for the laser pulse that has no chirp in the final stage. In addition to the high efficiency, the beam energy spread of <1% can be obtained owing to the DBL. The mechanism of how the DBL effect maintains a low energy spread will be elaborated on in the later section.

### DBL effect to reduce the beam energy spread

**Fig. 3.**
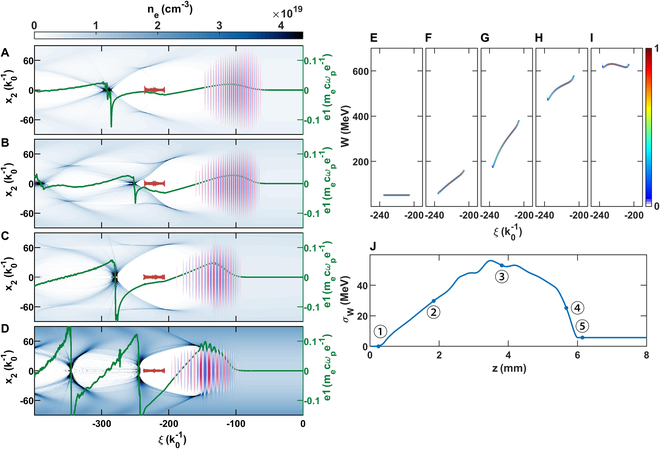
The on-axis electric field and the beam longitudinal phase space. (A to D) Snapshots of the density and the electric field distributions before/after the second (F and G) and third plateau (H and I), respectively. (E to I) Snapshots of beam longitudinal phase space corresponding to the tagged points in (J). (J) Absolute beam energy spread through the tri-plateau structure.

The use of stepwise plasma density plateaus connected with density up-ramps not only significantly increases the beam energy gain but also is capable of accelerating a large amount of beam charge with an extremely small energy spread through a DBL effect. The beam loading effect can be understood by tracking the accelerating field felt by the beam [13]. In Fig. 3A to D, the laser field, lineout of the accelerating field (green line), plasma, and beam density are plotted at the start to end points of each transition section (point a to point d) in Fig. 1B. In Fig. 3E to I, the beam energy versus axial position is shown at the same propagation distances. It can be seen that the plasma wake is overloaded ($\frac{dE_{z}}{\mathrm{d\xi}}<0$) during the first and at the entrance of the second plateau (Fig. 3A and B), leading to a negative energy chirp in the electron beam. On the other hand, in the third plateau, and during the exit of the second and third plateaus, the wake is underloaded ($\frac{dE_{z}}{\mathrm{d\xi}}>0$) (Fig. 3C and D) such that the energy chirp caused by the first plateau is almost perfectly removed. The transition from an over- to underloaded wake happens in the second plateau (between Fig. 3B and C).

This mechanism can be qualitatively described by the nonlinear theory of the blowout regime [38]. We find that the gradient of *E_z_* (see Materials and Methods) can be expressed as

1EpdEzdξ≈−2λkprb2+kp2 (1)

where $\lambda =k_{p}^{2}\int_{0}^{+\infty }\frac{n_{b}}{n_{p}}\mathrm{rdr}$ is the normalized linear charge density of the beam, $E_{p}\equiv \frac{m_{e}c\omega_{p}}{e}$ is the characteristic electric field in the plasma, and *r_b_* is the blowout radius. The linear charge density *λ*, which is independent of plasma density, can be assumed to not depend on the propagation distance. However, the value of *r_b_* at the location of the beam changes (increases) from dephasing within an individual plateau, and *k_p_* increases when crossing into the next plateau. For the parameters simulated, during the first plateau, the dominant term is $-\frac{2\lambda }{k_{p}r_{b}^{2}}$, while it becomes $\frac{k_{p}}{2}$ in the third plateau. In the second plateau, the dominant term changes from $-\frac{2\lambda }{k_{p}r_{b}^{2}}$ to $\frac{k_{p}}{2}$ as *r_b_* increases because of the increased plasma density and dephasing. The final energy chirp is obtained by integrating Eq. 3 with respect to the propagation distance, and eventually vanishes at the optimal distance (Fig. 3I). As seen in Fig. 3E to I, the beam energy chirp increases first and then decreases, and the residual energy chirp is almost fully compensated, which agrees with the analysis above. The final relative energy spread reaches as low as 0.83% (FWHM), while a considerable beam charge of 125 pC is accelerated.

### Scalability of the scheme guided by the LWFA scaling law

Assuming a fixed *a*_0_ and *λ*_0_, and that the laser is guided by a channel, the key physics can be viewed approximately self-similar, and scaling laws can be straightforwardly obtained (see Materials and Methods). When the plasma density changes from *n_p_* to *n*^′^*_p_*, the characteristic lengths of the key physics can be scaled by a factor $F=\sqrt{n_{p}/n_{p}^{\prime }}$. If we proportionally scale the focal waist and pulse length of the laser through $w_{0}^{\prime }=F\cdot w_{0}$ and *τ*′  = *F* · *τ*, the acceleration gradient, maximum beam energy gain, and the number of electrons that can be accelerated scale as *E_z_*′  = *F*^−1^*E_z_*, 〈∆*W*〉′  = *F*^2^ · 〈∆*W*〉, and *N*′ = *F* · *N*, respectively, according to the scaling law of LWFAs [40] (see Materials and Methods for the derivation). The energy transfer efficiency *η*′ is an invariant under this scaling since.

$$\eta^{\prime }=\frac{N^{\prime }\cdot {\langle ∆W\rangle }^{\prime }}{W_{\text{laser}}^{\prime }}=\frac{\mathrm{FN}\cdot F^{2}\langle ∆W\rangle }{F^{3}W_{\text{laser}}}=\eta$$ (2)

**Fig. 4.**
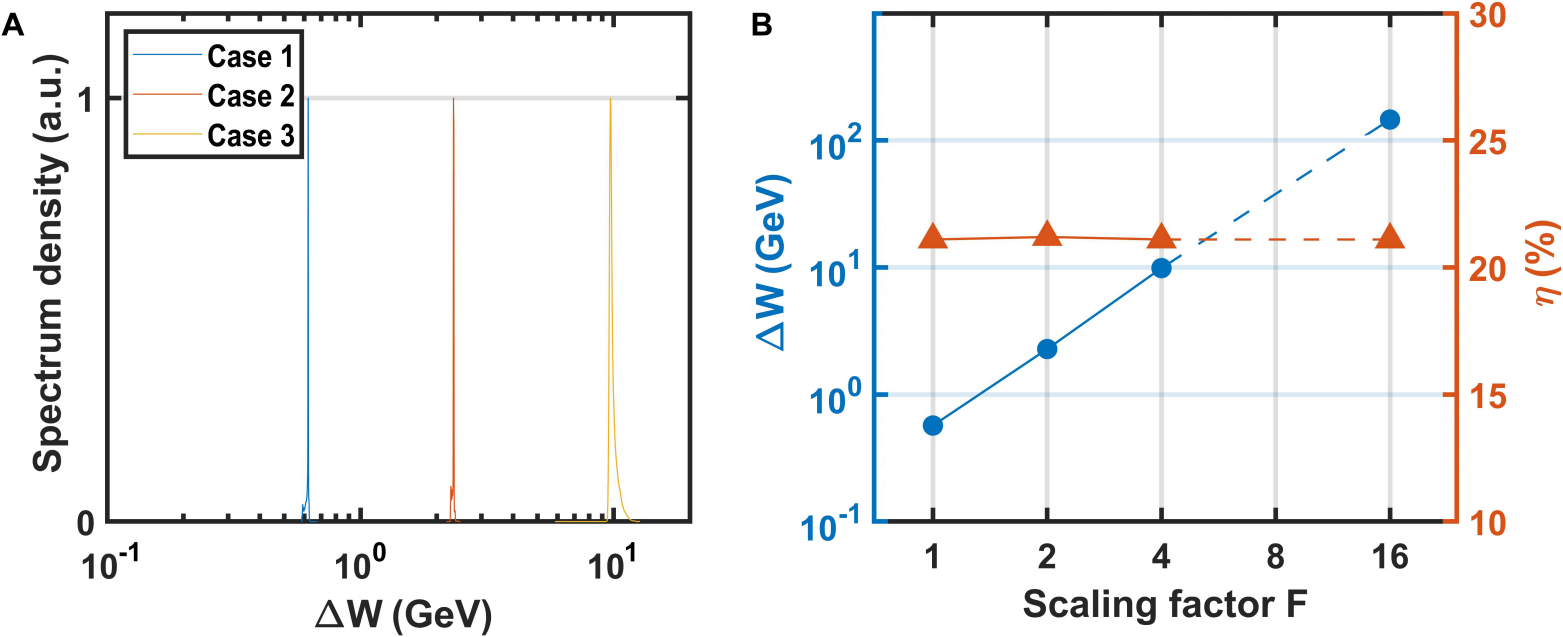
The energy gain and spectra of the scaled cases. (A) Energy spectra and (B) averaged beam energy gain and transfer efficiency of cases 1 to 3 in Table 1.

We carried out simulations with *F* = 2 and *F* = 4 to validate the scaling law, and the results are summarized in Table 1. In these simulations, the initial parameters of laser, plasma, and electron beam are scaled from case 1 and the results (energy gain, efficiency) show excellent agreement with what the scaling law predicts as shown in Fig. 4B. In each case, the final energy spread remains below 1%. Case 3 corresponds to a 500-pC electron beam being accelerated to ~10 GeV by a 22-J laser pulse, which is within the capability of modern petawatt lasers. The calculated energy transfer efficiency greatly exceeds the results in previous LWFA experiments and simulations [45,46]. Experimental verification of case 3 can be attempted in the near term. Furthermore, we can extrapolate the proposed scheme to *F* = 16 in column 4. In this case, an LWFA driven by a 1.4-kJ, 340-fs laser pulse would accelerate a 2-nC electron beam with ~150-GeV energy with an average acceleration gradient of 6 GeV/m. Laser with such energy and beams with such charge are already available. The proposed scheme could thus provide the electron arm of a Higgs factory, with ~20% efficiency and sub-1% energy spread. For comparison, a scaled case (case 5) using a uniform plasma channel is also listed in Table 2. In this case, only about one-third of the energy gain in case 4 is achieved, and the energy spread grows up to 15%. To scale the scheme to even higher energy approaching 10 TeV, in-depth studies on the limitations caused by physics such as ion motion should be conducted in the future.

**Table 1.** Parameter designs of cases 1 to 4 for *a*_0_ = 2 and *λ*_0_ = 800 nm according to the scaling law, in which cases 1 to 3 are PIC simulation results and case 4 is an extrapolation to obtain the 145.9-GeV electron beam. As a comparison, case 5 uses the same laser pulse as case 4 but in a matched uniform plasma channel, which is scaled from the case of the yellow dashed lines in Fig. 1C.

Case	1	2	3	4	5 (uniform channel)
Scaling factor *F*	1	2	4	16	16
Averaged energy gain (GeV)	0.57	2.28	9.88	145.9	51
Laser peak power (TW)	15.3	61.2	245	3,917	3,917
Pulse length (fs)	21.5	43.0	86.0	344	344
*w*_0_ (μm)	10.7	21.3	43.0	171	171
Laser pulse energy (J)	0.34	2.76	22.1	1,393	1,393
Plasma density of first plateau (cm^−3^)	2 × 10^18^	5 × 10^17^	1.25 × 10^17^	7.81 × 10^15^	7.81 × 10^15^
Plasma length (mm)	6.1	48.8	390	2.5e4	3.3e4
Electron beam charge (pC)	125	250	492	2,000	2,000
Energy spread (FWHM)	0.42%	0.65%	0.63%	<1%	~15%
Energy efficiency	21.1%	21.2%	21.1%	~21.1%	~7.4%

**Table 2.** Scaling of the laser, plasma, and beam parameters with scaling factor *F* for fixed *a*_0_ and *λ*_0_

	Formula	Scaled parameters for fixed *a*_0_, *λ*_0_ with scaling factor *F*
Laser	$k_{p}w_{0}=2\sqrt{a_{0}}$	$w_{0}^{\prime }=F\cdot w_{0}$
	*cτ* = *w*_0_	*τ*′ = *F* · *τ*
Plasma	$k_{p}^{-1}\propto 1/\sqrt{n_{p}}$	$n_{p}^{\prime }=F^{-2}\cdot n_{p}$
	$k_{p}R=2\sqrt{a_{0}}$	*R*′ = *F* · *R*
	$L_{\phi }=\frac{3}{4}\frac{k_{0}^{2}}{k_{p}^{2}}\frac{\sqrt{a_{0}}}{k_{p}}$	$L_{\phi }^{\prime }=F^{3}\cdot L_{\phi }$
Beam	$\langle \text{∆}W\rangle =\frac{2}{3}m_{e}c^{2}\frac{k_{0}^{2}}{k_{p}^{2}}a_{0}$	〈∆*W*〉′ = *F*^2^ · 〈∆*W*〉
	$N=\frac{8}{15k_{0}r_{e}}\sqrt{\frac{P}{m_{e}^{2}c^{5}/e^{2}}}$	*N*′ = *F* · *N*
	*σ_z_* ∝ *R*	$\sigma_{z}^{\prime }=F\cdot \sigma_{z}$

## Materials and Methods

### High-fidelity PIC simulation

The propagation and the evolution of the laser pulse are of crucial importance in LWFA research. Thus, high-fidelity modeling of the laser–plasma interaction including nonlinear dispersion effects is critical for the calculation of energy gain and energy transfer efficiency. Standard PIC simulations utilize a second-order Yee solver to solve the electro-magnetic fields, which could suffer from significant numerical dispersion, numerical Cerenkov radiation (NCR), and self-fields from the beam for the propagation distances considered here. These numerical errors may cause the PIC simulations to overestimate the dephasing effect that is closely related to the energy efficiency from laser pulse to the electron beam, and the growth of the emittance and energy spread of the beams. In this article, a customized finite difference EM solver, which can eliminate the numerical dispersion, NCR, and self-forces, is adopted [47–50].

All the simulations are conducted using the quasi-three-dimensional (3D) version of the PIC code OSIRIS. For the *F* = 1 case, the simulation window has a dimension of $150k_{0}^{-1}\times 400k_{0}^{-1}\;$ with 500 × 2,000 cells in the *r* and *z* directions, respectively. This corresponds to cell sizes of $\Delta r=0.30k_{0}^{-1}$ and $\Delta z=0.20k_{0}^{-1}$ (where the wave number *k*_0_ = 2*π*/*λ*_0_ and the wavelength *λ*_0_ = 800 nm). The time step is chosen as $\text{∆}t=0.1c^{-1}k_{0}^{-1}$. In the scaled cases of *F* = 2 and *F* = 4, the sizes of the simulation window and transverse cell size are scaled accordingly, while the longitudinal cell size is kept at $\Delta z=0.20k_{0}^{-1}$. There are 10 macroparticles in each cell and 8 duplications in the azimuthal direction. Only the *m* = 0 and *m* = 1 azimuthal modes are kept in the simulations.

### Waveform evolution of the nonlinearly chirped laser pulse

Due to the self-phase modulation of the laser pulse in the plasma, the laser pulse loses its energy by the frequency downshifting (photon deceleration) with the rate expressed as [51–53]:

$$r_{\mathrm{ph}}\equiv \frac{1}{k}\frac{\partial k}{\partial t},$$ (3)

where *k* is the local wave number of the laser pulse. This photon deceleration rate has a distribution along *ξ* (the inset of Fig. 2B) and is almost nonevolving during most of its propagation length. In the proposed high-efficiency scheme, the nonlinear chirp is added to the laser pulse to offset this photon deceleration. Another option is to shape the pulse to provide a wake with a constant photon deceleration rate throughout the entire pulse [54].

**Fig. 5.**
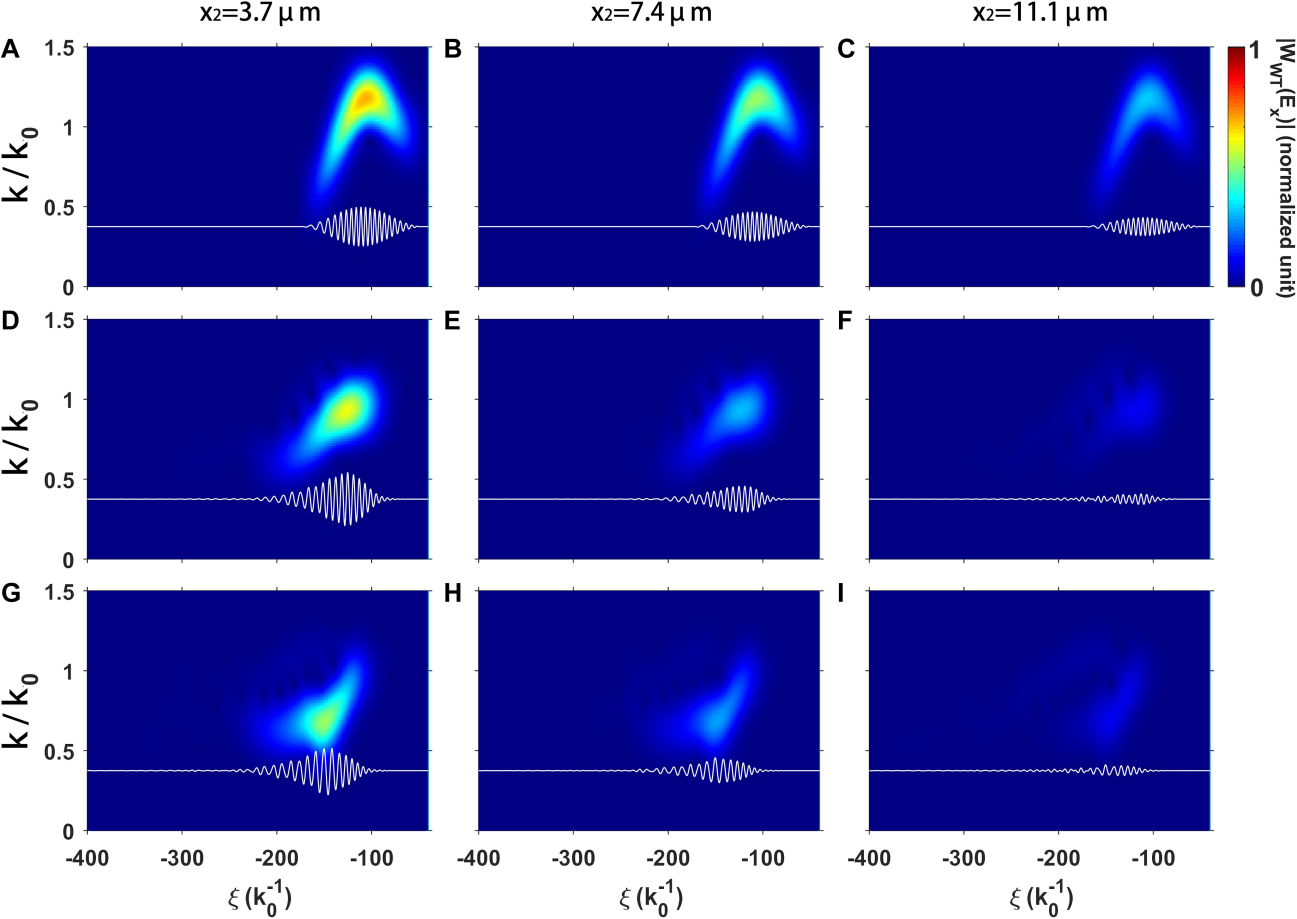
(A to I) Off-axis spectrograms (in color-obtained by Wigner-wavelet transforming the electric field) of the nonlinearly chirped laser pulse at various times propagating through a single plateau with different transverse positions. The first, second, and third rows show the spectrograms at *z* = 0, *z* = 7.39*z*_*R*_, and *z* = 11.36*z*_*R*_, where the Rayleigh length *z*_*R*_ of the original laser pulse is 447 μm. The different columns show the spectrograms for different transverse positions with* x*_*2*_* *= 3.7 μm, 7.4 μm, and 11.1 μm. Other parameters of the wavelet transform are the same with Fig. 2. The white lines are the corresponding lineouts of laser transverse electric field.

In addition to the on-axis waveform evolution of the nonlinearly chirped pulse (Fig. 2), off-axis waveform evolutions with different transverse position up to 11.1 μm are provided in Fig. 5 for integrality, noting that the matched *w*_0_ = 10.7 μm. Each row shows the laser time–frequency distribution at a different time when the laser pulse propagates 0, 7.39*z_R_* and 11.36*z_R_*, with *z_R_* = 447 μm is the Rayleigh length, while each column shows the spectrograms with different transverse positions at *x*_2_ = 3.7 μm, 7.4 μm, and 11.1 μm, noting that the beam waist of the initial laser pulse *w*_0_ = 10.7 μm. The off-axis waveforms show similar evolution with the on-axis waveform (Fig. 2B, D, and F).

### Calculation of the gradient of *E_z_*

To derive the expression of *E_z_*, the nonlinear theory of the bubble regime [38] is followed here. In the ultra-relativistic limit where the maximum blowout radius *k_p_R_b_* ≫ 1, the trajectory of the innermost particle is given by

$$r_{b}\frac{d^{2}r_{b}}{d\xi^{2}}+2{\left(\frac{dr_{b}}{\mathrm{d\xi}}\right)}^{2}+1=\frac{4\lambda \left(\xi \right)}{k_{p}^{2}r_{b}^{2}}$$ (4)

where *r_b_*(*ξ*) is the radial position of the innermost particle in the bubble sheath and $\lambda =k_{p}^{2}\int_{0}^{+\infty }n_{b}/n_{p}\mathrm{rdr}$ is the normalized linear charge density of the electron bunch. The longitudinal electric field *E_z_* can be expressed as a function of *r_b_*[40],

$$\frac{E_{z}}{E_{p}}=\frac{1}{E_{p}}\frac{\partial \psi }{\partial \xi }\approx -\frac{k_{p}}{2}r_{b}\frac{dr_{b}}{d\xi }$$ (5)

where *ψ* ≡ *ϕ* − *A_z_*/*c* is the pseudo-potential of the plasma wake. The gradient of the accelerating field *dE_z_*/*dξ* is obtained via differential of Eq. 5,

$$\begin{array}{c} \frac{1}{E_{p}}\frac{dE_{z}}{\mathrm{d\xi}}\approx -\frac{k_{p}}{2}{\left(\frac{dr_{b}}{\mathrm{d\xi}}\right)}^{2}-\frac{k_{p}}{2}r_{b}\frac{d^{2}r_{b}}{d\xi^{2}}=\\ -\frac{2\lambda }{k_{p}r_{b}^{2}}+\frac{k_{p}}{2}+\frac{k_{p}}{2}{\left(\frac{dr_{b}}{d\xi }\right)}^{2}\approx -\frac{2\lambda }{k_{p}r_{b}^{2}}+\frac{k_{p}}{2} \end{array}$$ (6)

In Eq. 6, the term $\frac{k_{p}}{2}{\left(\frac{dr_{b}}{d\xi }\right)}^{2}$ is omitted since it is negligible for most part of the bubble.

### Scaling of the simulations

The initial parameters for the scaled simulations are guided by LWFA scaling laws [40], starting from the scaling of the accelerating wakefield structure. For convenience, real and normalized units are used in this section. Through simulations, it has been found that a relatively stable propagation of the ion cavity is realized when $k_{p}w_{0}≃k_{p}R_{b}≃2\sqrt{a_{0}}$, where *w*_0_ is the initial laser beam waist and *R_b_* is the blowout radius of the ion cavity [38,39]. For the parameters being considered here, these relationships hold in a parabolic density channel if the density at the bottom of the channel is used. The connection between the plasma density and the size of the ion cavity is established as *n_p_* ∝ *F*^−2^ because $k_{p}\propto \sqrt{n_{p}}$, where *F* is the scaling factor of the radius of the ion cavity. To efficiently drive the wake, the laser pulse length should fill the first half of the ion cavity; thus, *cτ*_FWHM_ ∝ *R_b_*, where *τ*_FWHM_ is the pulse duration. Hence, the laser pulse energy scales with *F*^3^. The length of the plasma structure *L_acc_* is characterized by the dephasing length $L_{\phi }=\frac{4}{3}\frac{k_{0}^{2}}{k_{p}^{2}}\frac{\sqrt{a_{0}}}{k_{p}}$, providing *L_acc_* ∝ *F*^3^. Using the scaling law of the accelerating field, $E_{z}\propto \frac{m_{e}c\omega_{p}}{e}\propto F$, the expected beam energy gain is obtained as 〈∆*W*〉 ∝ *F*^2^. Another initial parameter to be determined is the loaded electron number *N*, and based on [13,38,55], it should scale with the electron number that is expelled from the ion cavity, leading to $N\propto n_{p}R_{b}^{3}\propto F$. The relevant formulas and scaling factors are summarized in Table 2.

As for the PIC simulations, the simulation window should scale with the ion cavity, e.g., *r*_window_ ∝ *F* and *z*_window_ ∝ *F*. The transverse cell size ∆*r* scales with *R_b_* to resolve the plasma wavelength, while the longitudinal cell size ∆*z* is fixed to resolve the laser wavelength.

## Data Availability

The data that support the plots within this paper and other findings of this study are available from the corresponding author upon reasonable request.
